# Impact of marital status at diagnosis on survival and its change over time between 1973 and 2012 in patients with nasopharyngeal carcinoma: a propensity score‐matched analysis

**DOI:** 10.1002/cam4.1232

**Published:** 2017-10-16

**Authors:** Cheng Xu, Xu Liu, Yu‐Pei Chen, Yan‐Ping Mao, Rui Guo, Guan‐Qun Zhou, Ling‐Long Tang, Ai‐Hua Lin, Ying Sun, Jun Ma

**Affiliations:** ^1^ Department of Radiation Oncology Sun Yat‐sen University Cancer Center State Key Laboratory of Oncology in South China Collaborative Innovation Center for Cancer Medicine Guangzhou Guangdong Province China; ^2^ Department of Medical Statistics and Epidemiology School of Public Health Sun Yat‐sen University Guangzhou Guangdong Province China

**Keywords:** Marital status, nasopharyngeal carcinoma, propensity score‐matched analysis, SEER, survival

## Abstract

The impact of marital status at diagnosis on survival outcomes and its change over time in patients with nasopharyngeal carcinoma (NPC) are unclear. The Surveillance, Epidemiology, and End Results (SEER) database was used to identify patients diagnosed with NPC in the United States from 1973 to 2012. A primary comparison (married vs. unmarried) was implemented with 1:1 propensity score matching. Secondary comparisons were performed individually between three unmarried subgroups (single, separated/divorced, widowed) and married group. The effect of marital status on cause‐specific survival (CSS) and overall survival (OS) were evaluated using univariate/multivariate analysis. Moreover, we investigated the change over time (1973–2012) in the effect of marital status on NPC survival. Married patients had better 5‐year CSS/OS than unmarried patients (61.1% vs. 52.6%, *P *<* *0.001; 55.6% vs. 45.3%, *P *<* *0.001, respectively). In multivariate analysis, unmarried patients had significantly poorer CSS/OS than married patients (adjusted hazard ratio [aHR] = 1.35, *P *<* *0.001; aHR = 1.40, *P *<* *0.001, respectively). The survival benefit of being married was only detected in non‐Hispanic white and Chinese American patients. Single, separated/divorced, and widowed patients had significantly poorer CSS/OS than married patients (aHR = 1.37 and 1.37; 1.46 and 1.42; 1.43 and 1.48, respectively; all *P *<* *0.001). The change over time in the effect of marital status on survival was more stable in male than female. The strength of the negative effect of separated/divorced and widowed status showed a downward and upward trend, respectively. Gender difference in the adverse effect of single status on NPC survival became smaller over time. Only non‐Hispanic white and Chinese American patients with NPC obtain survival benefits from married status. Single and widowed patients are regarded as high‐risk population

## Introduction

Nasopharyngeal carcinoma (NPC) is a malignant head and neck cancer with an uneven global distribution; the highest incidences are observed in Southeast Asia, North Africa, the Middle East and Alaska 1. Radiotherapy and chemotherapy are widely used in the treatment of NPC and have satisfactory therapeutic efficacy. However, as a result of drug‐induced toxicity and the close proximity between the tumor and surrounding vital organs, patients may suffer severe adverse outcomes that significantly affect their daily activities, such as altered speech, erosion of the oral mucosa, visual impairment and mastication dysfunction 2, 3. Moreover, undergoing multiple courses of radiochemotherapy necessitates meticulous heath care and can be financially expensive. Therefore, social support is of great importance for patients with NPC.

Married status is regarded as a type of social support with general beneficial to all individuals (e.g., cancer‐free people and cancer patients), especially in the elderly 4. A previous study reported generally significant survival benefit for married patients compared to unmarried patients in analyses of the ten leading causes of cancer‐related deaths in the United States. Meanwhile, marriage conferred a greater survival benefit than the published survival benefits reported for chemotherapy in five of the ten leading cancers 5. Thus, the strength of the association between marriage and survival outcomes varies depending on the type of malignancy. Moreover, in studies that investigated the value of marital status on survival in a single malignancy, conflicting conclusions have been obtained with both positive 6, 7, 8 and nonsignificant results 9, 10.

Little is known about the effect of marital status on survival in patients with NPC. Although a recent study showed married status conferred a survival benefit among patients with head and neck cancers, this study did not compare married patients with specific subgroups of unmarried patients, namely, single, separated/divorced and widowed patients. Moreover, the short median follow‐up time (19 months) and generally unbalanced baseline characteristics of the groups indicate these results have relatively low validity 11. Besides, the effect of marital status on survival in cancer patients has been reported to change over time 12. Whether this change has a different trend in male and female patients with NPC is worthy of investigation.

In this study, we extracted detailed data on patients diagnosed with NPC in the United States between 1973 and 2012 from the Surveillance, Epidemiology, and End Results (SEER) database, and created well‐matched cohorts using the propensity score matching to investigate the effect of marital status on 5‐year survival outcomes of petients with NPC.

## Materials and Methods

### Data source and patient selection

We used the SEER database released in April, 2015 as the data source for this study. The SEER program is the only comprehensive source of national cancer data in the United States. Sponsored by the National Cancer Institute, the SEER program collects demographic, clinicopathologic, and survival data on a per‐patient basis from eighteen cancer registries (SEER‐18) across the continental United States, Hawaii and Alaska. All data generated during the period from 1973 to 2012 is recorded in the SEER database, with a follow‐up cutoff date of 31 December 2012. Although not all registries contributed cases throughout the entire period, the SEER‐18 covers 27.8% of the population in the US and has a typical distribution, and is therefore thought to represent the US population as a whole 13.

With the help of SEER*Stat software, version 8.2.1 (National Cancer Institute, Bethesda, MD), we obtained detailed data on patients diagnosed with NPC between 1973 and 2012 from the SEER‐18. We excluded cases using the following criteria: (1) age at diagnosis <18 years‐old or unknown; (2) follow‐up period <6 months; (3) cases not newly‐ or pathologically‐diagnosed; (4) unknown marital status or domestic partners; (5) lack of clear records on stage, histology and treatment strategy; (6) patients with prior malignancy. The key raw data has been uploaded onto the Research Data Deposit public platform, with the approval number as RDDA2017000129 14.

### Study variables and endpoints

Marital status was classified as married, single (never married), separated/divorced, or widowed; the latter three categories were combined as the unmarried. All marital status data was recorded at the time of diagnosis. Race/ethnicity was classified as non‐Hispanic white, non‐Hispanic black (determined by both of white/black race and non‐Hispanic ethnicity), Hispanic (determined only by Hispanic ethnicity), Chinese, or other (American Indian/Alaska Native, Asian/Pacific Islander, and unknown). Eighteen cancer registries were categorized into four regions – West, Northeast, North central, and South – according to the classification principles of the US Census Bureau 13. For the year of diagnosis, the period from 1973 to 2012 was divided into four decades.

Considering that different stage systems and editions were used to stage the patients with NPC over this long period of time, we adopted the SEER historic stage system in the study. This simplified stage system is applicable to all patients diagnosed between 1973 and 2012. According to the codes of the International Classification of Diseases for Oncology, 3rd edition (ICD‐O‐3), we classified histological type as keratinizing (8070 and 8071), differentiated non‐keratinizing (8072 and 8073), undifferentiated non‐keratinizing (8020, 8021 and 8082), or other (the remaining codes). We classified treatment strategies as definitive treatment (i.e., surgery and/or radiotherapy) and no definitive treatment, since the SEER database does not provide any information about chemotherapy or systemic therapy.

End‐points were cause‐specific survival (CSS; from diagnosis until death due to NPC or last date known alive) and overall survival (OS; to death due to any cause or last follow‐up, whichever happened first).

### Statistical analysis

All statictical methods were applied to the primary comparison (unmarried vs. married) and three secondary comparisons (single, separated/divorced and widowed vs. married, respectively). Follow‐up time was reported as median and interquartile range (IQR). Descriptive statistics provided as continuous variables were reported as means and standard deviations; categorical variables as frequencies and percentages. Baseline characteristics were analyzed using the Spearman test for constinuous data and chi‐square test for categorical measurements. The cumulative 5‐year CSS and OS were estimated using the Kaplan–Meier method and compared using the log‐rank test. Moreover, we implemented the univariate and multivariate Cox regression method to quantitatively determine the effect of marital status on survival; the multivariate analysis was adjusted for all baseline characteristics. For secondary comparisons, we generated a forest plot using Microsoft Excel (Microsoft Inc., Redmond, WA, USA) via Neyeloff's method 15 to summarize the adjusted hazard ratios (aHRs) and 95% confidence intervals (CIs) of three unmarried subgroups versus the married group. Based on 1‐to‐1 matched data sets, we calculated HRs and 95% CIs of the association between marital status and survival in each decade interval between 1973 and 2012, and developed line charts using Microsoft Excel to demonstrate the change over time in the effect of marital status on CSS/OS in male and female patients; HR >1 represents a survival benefit favoring married patients.

To mimic the randomized controlled trials and minimize the influence of potential confounders on selection bias, a 1‐to‐1 propensity score matching method without replacement was performed using the nearest‐neighbor method with a stringent caliper of 0.05 16. All variables for the previously listed baseline characteristics were entered into the model using a backward stepwise likelihood ratio algorithm. All statistical analyses and figures were generated from the matched datasets with SPSS, version 22.0 (SPSS Inc., Chicago, IL), unless otherwise specified. Two‐sided *P *< 0.05 were considered significanct.

## Results

### Patients identification and baseline characteristics

**Figure 1:**
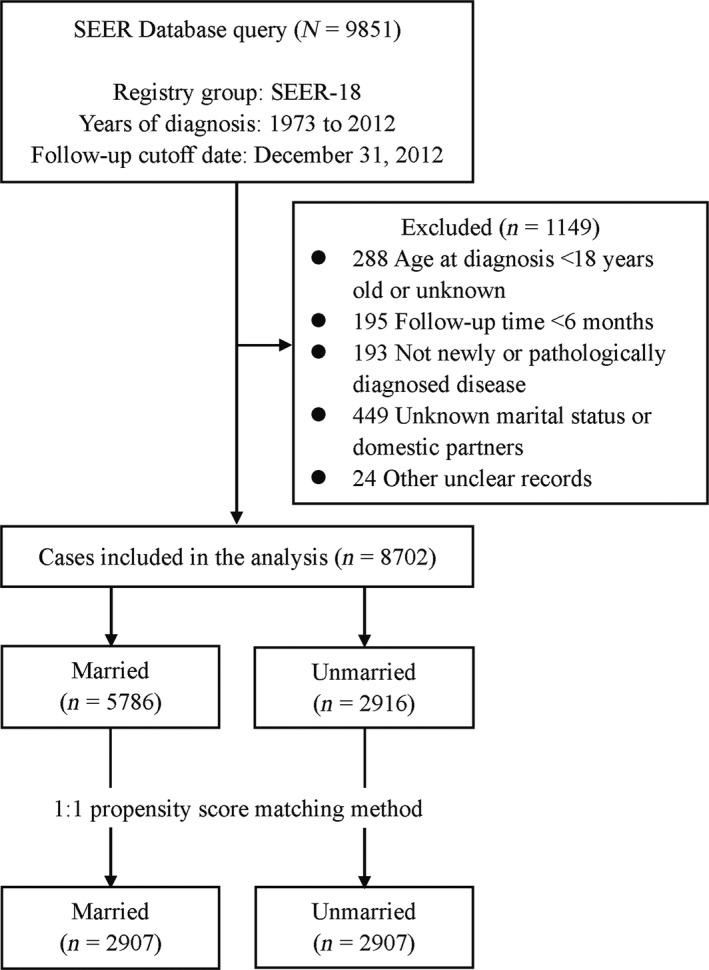
Flowchart describing the inclusion and exclusion of patients.

We extracted data on 9851 patients diagnosed with NPC between 1973 and 2012 from the SEER‐18. After the rounds of selection, 8702 eligible patients were included (Fig. 1). For the primary comparison, the original data set (*n = *8702) included 5786 married patients and 2916 unmarried patients; significant difference in all baseline characteristics was observed between the two groups (all *P *≤* *0.007; Table 1).

**Table 1:** Baseline characteristics of the married and unmarried patients with NPC in the original/matched data sets

Characteristics	Original data set			Matched data set		
	Married (*N = *5786) no. (%)^d^	Unmarried (*N = *2916) no. (%)^d^	*P*‐value	Married (*N = *2907) no. (%)^d^	Unmarried (*N = *2907) no. (%)^d^	*P*‐value
Follow‐up time			–			–
Median, months	146	136		132	136	
IQR, months	76–259	66–239		67–241	66–240	
No. of death at 5‐year			–			–
Cause‐specific	1984 (34.3)	1239 (42.5)		1022 (35.2)	1234 (42.4)	
Overall	2397 (41.4)	1508 (51.7)		1205 (41.5)	1500 (51.6)	
Age at diagnosis^a^			0.007			0.138
≤50 years	2260 (39.1)	1226 (42.0)		1280 (44.0)	1224 (42.1)	
Mean ± SD, years	41.65 ± 6.45	36.85 ± 9.84		41.42 ± 6.68	36.85 ± 9.84	
>50 years	3526 (60.9)	1690 (58.0)		1627 (56.0)	1683 (57.9)	
Mean ± SD, years	62.92 ± 8.64	65.13 ± 10.07		63.49 ± 8.75	65.11 ± 10.06	
Gender			<0.001			0.358
Male	4302 (74.4)	1791 (61.4)		1825 (62.8)	1791 (61.6)	
Female	1484 (25.6)	1125 (38.6)		1082 (37.2)	1116 (38.4)	
Race/ethnicity			<0.001			<0.001
Non‐Hispanic white	2303 (39.8)	1330 (45.6)		1433 (49.3)	1323 (45.5)	
Non‐Hispanic black	302 (5.2)	388 (13.3)		174 (6.0)	386 (13.3)	
Hispanic	341 (5.9)	254 (8.7)		177 (6.1)	254 (8.7)	
Chinese	1413 (24.4)	388 (13.3)		579 (19.9)	388 (13.3)	
Other^b^	1427 (24.7)	556 (19.1)		544 (18.7)	556 (19.1)	
Registry region^c^			<0.001			0.845
West	4123 (71.3)	1913 (65.6)		1924 (66.2)	1907 (65.6)	
Northeast	655 (11.3)	379 (13.0)		378 (13.0)	376 (12.9)	
North central	679 (11.7)	395 (13.5)		394 (13.6)	395 (13.6)	
South	329 (5.7)	229 (7.9)		211 (7.3)	229 (7.9)	
SEER historic stage			<0.001			0.464
Localized	700 (12.1)	282 (9.7)		293 (10.1)	282 (9.7)	
Regional	3300 (57.0)	1531 (52.5)		1566 (53.9)	1531 (52.7)	
Distant	1450 (25.1)	884 (30.3)		826 (28.4)	881 (30.3)	
Unstaged	336 (5.8)	219 (7.5)		222 (7.6)	213 (7.3)	
Histological type			<0.001			0.054
Keratinizing	2182 (37.7)	1203 (41.3)		1160 (39.9)	1198 (41.2)	
Differentiated non‐keratinizing	889 (15.4)	395 (13.5)		454 (15.6)	395 (13.6)	
Undifferentiated non‐keratinizing	1236 (21.4)	542 (18.6)		575 (19.8)	542 (18.6)	
Other	1479 (25.6)	776 (26.6)		718 (24.7)	772 (26.6)	
Year of diagnosis			<0.001			0.070
1973–1982	752 (13.0)	301 (10.3)		347 (11.9)	300 (10.3)	
1983–1992	891 (15.4)	427 (14.6)		390 (13.4)	427 (14.7)	
1993–2002	1798 (31.1)	894 (30.7)		841 (28.9)	890 (30.6)	
2003–2012	2345 (40.5)	1294 (44.4)		1329 (45.7)	1290 (44.4)	
Treatment			<0.001			0.162
Surgery and/or RT	5231 (90.4)	2481 (85.1)		2518 (86.6)	2481 (85.3)	
No definitive treatment	555 (9.6)	435 (14.9)		389 (13.4)	426 (14.7)	

NPC, nasopharyngeal carcinoma; IQR, interquartile range; SEER, Surveillance, Epidemiology, and End Results Program; no., number; SD, standard deviation; RT, radiotherapy.

a “Age at diagnosis” was analyzed as categorical measurement (age ≤50; age >50).

b “Other” includes American Indian/Alaska Native, Asian/Pacific Islander, and unknown.

c “West” includes Seattle‐Puget Sound, Greater California, San Francisco‐Oakland, San Jose‐Monterey, Los Angeles, Utah, New Mexico, Alaska, and Hawaii. “Northeast” includes Connecticut and New Jersey. “North central” includes Iowa and Detroit. “South” includes Kentucky, Atlanta, Rural Georgia, Greater Georgia, and Louisiana.

d Percentages may not add up to 100 because of rounding.

After application of the 1:1 propensity score matching method, the matched data set (*n = *5814) included 2907 married patients and 2907 unmarried patients. The median follow‐up time was 132 months (IQR = 67–241 months) for married patients and 136 months (IQR = 66–240months) for unmarried patients. All baseline characteristics were well‐matched between groups, except for race/ethnicity (*P *<* *0.001). Patients in the married group were more likely to be non‐Hispanic white and Chinese American. The features of the original/matched data set are summarized in Table 1. Moreover, in secondary comparisons, baseline characteristics of three matched cohorts (single, separated/divorced and widowed vs. married, respectively) were generally well balanced (Table S1).

### Effect of marital status on CSS and OS in the primary comparison

**Figure 2:**
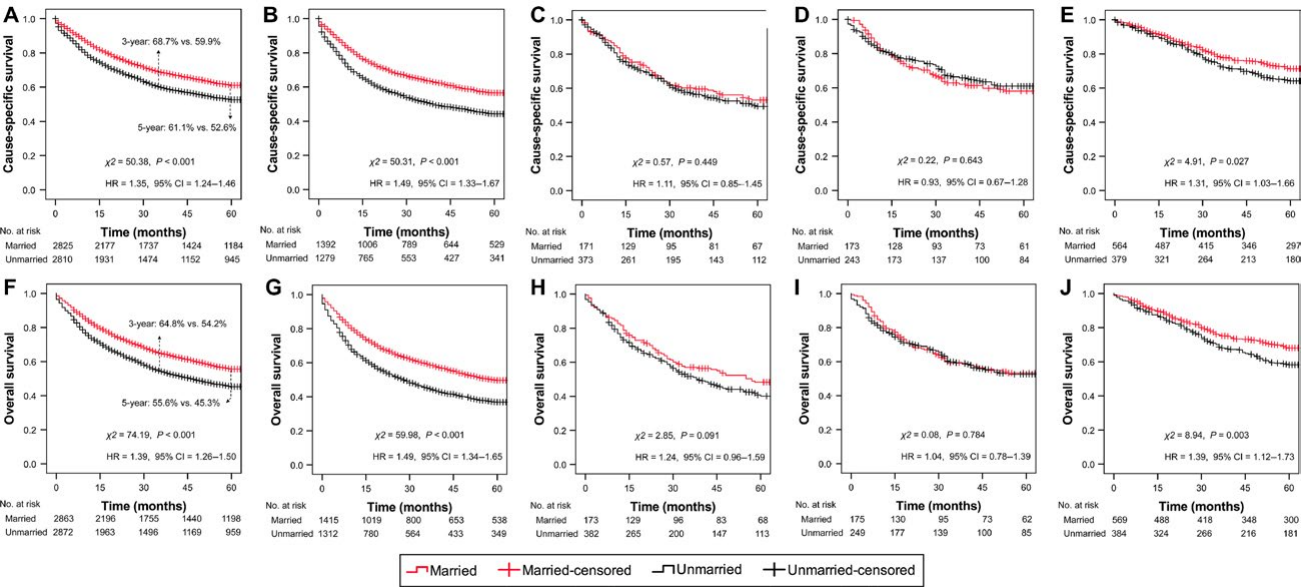
Kaplan–Meier survival curves in patients with NPC of different race/ethnicity. Survival curves for CSS (A–E) and OS (F–J) were stratified by marital status. (A, F) All races/ethnicities; (B, G) Non‐Hispanic white; (C, H) Non‐Hispanic black; (D, I) Hispanic; (E, J) Chinese. CSS, cause‐specific survival; OS, overall survival; HR, hazard ratio; CI, confidence interval; No., number; NPC, nasopharyngeal carcinoma.

The 5‐year cumulative CSS/OS rates were 61.1% and 55.6% for married patients, and 52.6% and 45.3% for unmarried patients, respectively. Married patients (as reference) had better survival outcomes than unmarried patients (CSS: HR = 1.35, 95% CI = 1.24–1.46, *P *<* *0.001, Fig. 2A; OS: HR = 1.39, 95% CI = 1.26–1.50, *P *<* *0.001; Fig. 2F).

In univariate analysis, all baseline characteristics were identified as significantly predictive factors for CSS/OS, aside from non‐Hispanic black patients (Table 2). After adjustment in multivariate analysis, all aforementioned variables retained independent significance in CSS/OS, except for being non‐Hispanic black/Hispanic (all *P *≥* *0.072), residing in the Northeast/North central regions (all *P *≥* *0.117), and being diagnosed between 1983 and 1992 (*P *≥* *0.129). Unmarried status had a validated negative effect on survival outcomes compared to married status (CSS: aHR = 1.35, 95% CI = 1.24–1.47, *P *<* *0.001; OS: aHR = 1.40, 95% CI = 1.29–1.51, *P *<* *0.001). Moreover, Chinese American patients, female patients and patients diagnosed between 1993 and 2012 were more likely to have improved CSS/OS compared to their corresponding references (Table 2).

**Table 2:** Univariate and multivariate analysis of the effect of marital status on survival outcomes in NPC

Variables	CSS				OS			
	Univariate analysis		Multivariate analysis		Univariate analysis		Multivariate analysis	
	HR (95% CI)	*P‐*value	aHR (95% CI)^a^	*P‐*value	HR (95% CI)	*P‐*value	aHR (95% CI)^a^	*P‐*value
Age at diagnosis								
≤50 years	Reference		Reference		Reference		Reference	
>50 years	1.84 (1.68–2.01)	<0.001	1.63 (1.49–1.78)	<0.001	2.05 (1.89–2.22)	<0.001	1.84 (1.69–2.00)	<0.001
Gender								
Male	Reference		Reference		Reference		Reference	
Female	0.85 (0.78–0.92)	<0.001	0.88 (0.81–0.96)	0.005	0.88 (0.81–0.95)	0.001	0.90 (0.83–0.97)	0.006
Race/ethnicity								
Non‐Hispanic white	Reference		Reference		Reference		Reference	
Non‐Hispanic black	0.94 (0.82–1.08)	0.403	0.92 (0.80–1.05)	0.216	0.96 (0.85–1.08)	0.500	0.93 (0.82–1.06)	0.282
Hispanic	0.74 (0.63–0.88)	0.001	0.85 (0.72–1.01)	0.072	0.76 (0.65–0.89)	<0.001	0.91 (0.78–1.07)	0.247
Chinese	0.50 (0.44–0.57)	<0.001	0.66 (0.58–0.76)	<0.001	0.49 (0.44–0.55)	<0.001	0.69 (0.60–0.78)	<0.001
Other	0.60 (0.54–0.68)	<0.001	0.72 (0.63–0.81)	<0.001	0.59 (0.53–0.66)	<0.001	0.74 (0.66–0.84)	<0.001
Registry region								
West	Reference		Reference		Reference		Reference	
Northeast	1.21 (1.07–1.36)	0.003	1.01 (0.89–1.15)	0.843	1.21 (1.08–1.36)	0.001	1.01 (0.90–1.14)	0.836
North central	1.38 (1.23–1.56)	<0.001	1.04 (0.91–1.18)	0.578	1.47 (1.32–1.63)	<0.001	1.10 (0.98–1.23)	0.117
South	1.35 (1.16–1.56)	<0.001	1.21 (1.04–1.42)	0.017	1.34 (1.17–1.54)	<0.001	1.21 (1.04–1.39)	0.011
SEER historic stage								
Localized	Reference		Reference		Reference		Reference	
Regional	1.54 (1.30–1.84)	<0.001	1.78 (1.50–2.13)	<0.001	1.37 (1.18–1.60)	<0.001	1.62 (1.39–1.88)	<0.001
Distant	2.64 (2.21–3.15)	<0.001	3.26 (2.72–3.91)	<0.001	2.28 (1.95–2.66)	<0.001	2.86 (2.44–3.35)	<0.001
Unstaged	2.18 (1.76–2.70)	<0.001	1.63 (1.31–2.02)	<0.001	1.92 (1.59–2.32)	<0.001	1.46 (1.21–1.77)	<0.001
Histological type								
Keratinizing	Reference		Reference		Reference		Reference	
Differentiated non‐keratinizing	0.52 (0.46–0.60)	<0.001	0.66 (0.58–0.76)	<0.001	0.53 (0.47–0.60)	<0.001	0.68 (0.60–0.77)	<0.001
Undifferentiated non‐keratinizing	0.43 (0.38–0.48)	<0.001	0.54 (0.47–0.62)	<0.001	0.43 (0.39–0.49)	<0.001	0.57 (0.50–0.64)	<0.001
Other	0.67 (0.60–0.74)	<0.001	0.76 (0.68–0.84)	<0.001	0.68 (0.62–0.75)	<0.001	0.79 (0.71–0.86)	<0.001
Year of diagnosis								
1973–1982	Reference		Reference		Reference		Reference	
1983–1992	0.86 (0.74–0.99)	0.037	0.91 (0.89–1.05)	0.213	0.85 (0.74–0.97)	0.014	0.90 (0.79–1.03)	0.129
1993–2002	0.63 (0.55–0.72)	<0.001	0.75 (0.65–0.85)	<0.001	0.62 (0.55–0.70)	<0.001	0.74 (0.66–0.84)	<0.001
2003–2012	0.58 (0.51–0.66)	<0.001	0.52 (0.46–0.59)	<0.001	0.58 (0.51–0.65)	<0.001	0.53 (0.47–0.59)	<0.001
Treatment								
Surgery and/or RT	Reference		Reference		Reference		Reference	
No definitive treatment	2.79 (2.53–3.09)	<0.001	2.81 (2.53–3.12)	<0.001	2.67 (2.43–2.92)	<0.001	2.71 (2.46–2.98)	<0.001
Marital status								
Married	Reference		Reference		Reference		Reference	
Unmarried	1.35 (1.24–1.46)	<0.001	1.35 (1.24–1.47)	<0.001	1.39 (1.29–1.50)	<0.001	1.40 (1.29–1.51)	<0.001

NPC, nasopharyngeal carcinoma; CSS, cause‐specific survival; OS, overall survival; aHR, adjusted hazard ratio; CI, confidence interval; RT, radiotherapy; SEER, Surveillance, Epidemiology, and End Results Program.

a aHR was adjusted for demographics (age at diagnosis, gender, race/ethnicity), registry region, SEER historic stage, histological type, year of diagnosis, and treatment.

### Subgroup analysis of the primary comparison

Considering the unbalanced distribution of race/ethnicity between two groups in the matched data set of the primary comparison, we performed subgroup analysis based on race/ethnicity. Kaplan–Meier survival curves for CSS (Fig. 2B–E) and OS (Fig. 2G–J) revealed that only non‐Hispanic white patients (HR = 1.49, *P *<* *0.001; HR = 1.49, *P *<* *0.001, respectively) and Chinese patients (HR = 1.31, *P = *0.027; HR = 1.39, *P = *0.003, respectively) obtained a significant survival benefit from married status, while equivalent survival outcomes were observed between married and unmarried non‐Hispanic black patients, Hispanic patients, and patients with other races/ethnicities (Fig. S1). In multivariate analysis, the validated protective effects of married status on CSS/OS were only validated in non‐Hispanic white (aHR = 1.49, *P *<* *0.001; aHR = 1.48, *P *<* *0.001, respectively) and Chinese American patients (aHR = 1.29, *P = *0.041; aHR = 1.38, *P = *0.005, respectively, Table 3).

**Table 3:** Subgroup analysis of the effect of marital status on survival outcomes in NPC

Subgroups	CCS		OS	
	aHR (95% CI)^a^^,^ b	*P‐*value	aHR (95% CI)^a^^,^ b	*P‐*value
Race/ethnicity				
Non‐Hispanic white	1.49 (1.33–1.67)	<0.001	1.48 (1.33–1.64)	<0.001
Non‐Hispanic black	1.11 (0.84–1.47)	0.449	1.22 (0.95–1.57)	0.126
Hispanic	0.97 (0.69–1.36)	0.875	1.11 (0.82–1.50)	0.501
Chinese	1.29 (1.01–1.64)	0.041	1.38 (1.10–1.72)	0.005
Other	1.21 (0.98–1.50)	0.079	1.23 (1.01–1.50)	0.041
Age at diagnosis				
≤50 years	1.24 (1.07–1.44)	0.004	1.26 (1.10–1.45)	0.001
>50 years	1.38 (1.25–1.53)	<0.001	1.43 (1.30–1.57)	<0.001
Gender				
Male	1.35 (1.22–1.50)	<0.001	1.43 (1.29–1.57)	<0.001
Female	1.31 (1.13–1.51)	<0.001	1.30 (1.14–1.49)	<0.001
SEER historic stage				
Localized	1.67 (1.20–2.34)	0.003	1.66 (1.24–2.22)	0.001
Regional	1.37 (1.21–1.54)	<0.001	1.41 (1.26–1.57)	<0.001
Distant	1.34 (1.16–1.55)	<0.001	1.42 (1.25–1.63)	<0.001
Unstaged	1.10 (0.83–1.47)	0.496	1.01 (0.78–1.32)	0.935
Histological type				
Keratinizing	1.43 (1.27–1.61)	<0.001	1.45 (1.30–1.62)	<0.001
Differentiated non‐keratinizing	1.50 (1.17–1.92)	0.001	1.56 (1.25–1.96)	<0.001
Undifferentiated non‐keratinizing	1.40 (1.11–1.76)	0.004	1.44 (1.17–1.77)	0.001
Other	1.11 (0.93–1.31)	0.253	1.16 (0.99–1.35)	0.066
Treatment				
Surgery and/or radiotherapy	1.29 (1.18–1.42)	<0.001	1.36 (1.25–1.48)	<0.001
No definitive treatment	1.54 (1.28–1.84)	<0.001	1.46 (1.23–1.73)	<0.001
Year of diagnosis				
1973–1982	1.25 (0.99–1.56)	0.056	1.16 (0.95–1.41)	0.146
1983–1992	1.53 (1.25–1.87)	<0.001	1.62 (1.34–1.95)	<0.001
1993–2002	1.22 (1.05–1.43)	0.012	1.26 (1.09–1.45)	0.002
2003–2012	1.40 (1.22–1.60)	<0.001	1.43 (1.26–1.63)	<0.001

NPC, nasopharyngeal carcinoma; CSS, cause‐specific survival; OS, overall survival; aHR, adjusted hazard ratio; CI, confidence interval; SEER, Surveillance, Epidemiology, and End Results Program.

a “Married” was used as reference compared to “unmarried”.

b aHR was adjusted for demographics (age at diagnosis, gender, race/ethnicity), registry region, SEER historic stage, histological type, year of diagnosis, and treatment, except for the variable that is being analyze.

Further analyses were performed stratified by age at diagnosis, gender, SEER historic stage, histological type, treatment and year of diagnosis. Married status was associated with a protective effect on CSS/OS in all subgroups, except unstaged cases, patients with “other” histological type and patients diagnosed between 1973 and 1982 (Table 3).

### Effect of marital status on CSS and OS in the secondary comparison

**Figure 3:**
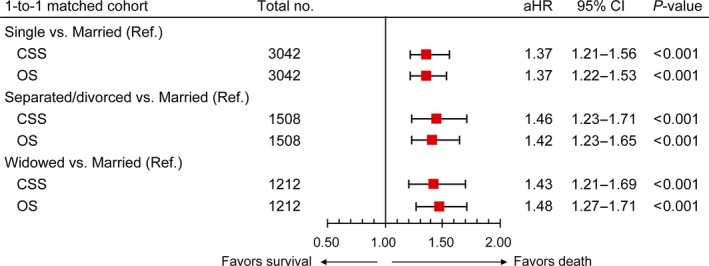
The effect of marital status on CSS and OS in the secondary comparison. Squares represent the aHRs with the 95% CIs indicated by horizontal bars. CSS, cause‐specific survival; OS, overall survival; aHR, adjusted hazard ratio; CI, confidence interval; no., number; Ref., reference.

A forest plot was used to assess the effect of marital status on CSS/OS in three 1‐to‐1 matched cohorts in the secondary comparison, namely, single versus married (*n = *3042; 1521 vs. 1521), separated/divorced versus married (*n = *1508; 754 vs. 754), and widowed versus married (*n = *1212; 606 vs. 606). Single patients (CSS: aHR = 1.37, *P *<* *0.001; OS: aHR = 1.37, *P *<* *0.001), separated/divorced patients (CSS: aHR = 1.46, *P *<* *0.001; OS: aHR = 1.42, *P *<* *0.001) and widowed patients (CSS: aHR = 1.43, *P *<* *0.001; OS: aHR = 1.48, *P *<* *0.001) were more likely to have poorer survival outcomes compared to married patients (Fig. 3).

### Change over time between 1973 and 2012 in the effect of marital status on survival

**Figure 4:**
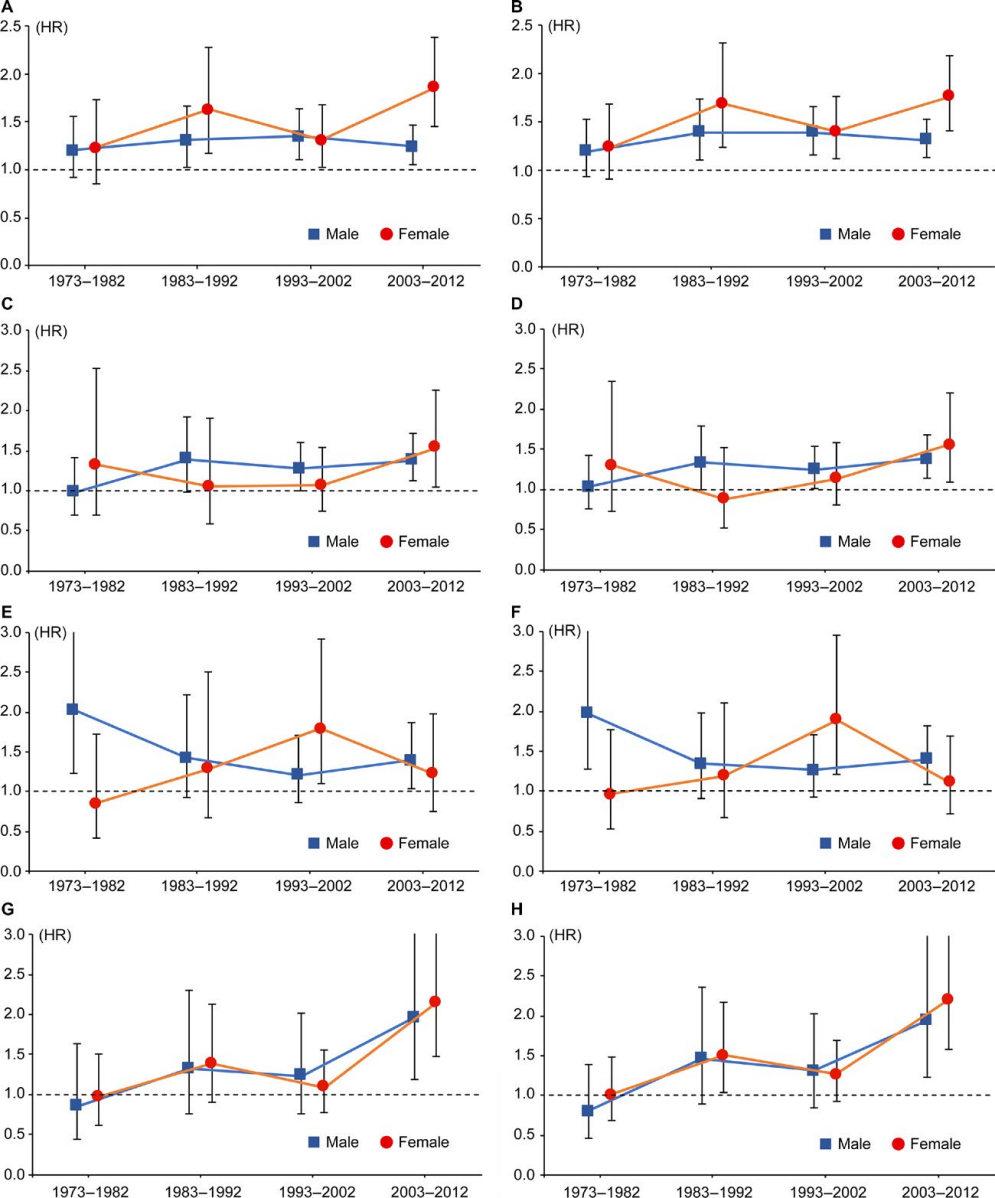
Change over time in the effect of marital status on survival outcomes. The *left* and *right* panel represent the change in the effect of marital status on CSS (A, C, E, G) and OS (B, D, F, H), respectively. Squares and circles represent the HRs of the association between marital status (unmarried vs. married [A, B]; single vs. married [C, D]; separated/divorced vs. married [E, F]; widowed vs. married [G, H]) and survival in male and female patients, respectively. 95% CIs were indicated by vertical bars. CSS, cause‐specific survival; OS, overall survival; HR, hazard ratio; CI, confidence interval.

As shown in Figures 4A–B, the change over time in the negative effect of unmarried status on CSS/OS was more stable in male patients than female patients. After 1982, the survival advantage of being married continued to exist in both male and female patients. Gender difference in the adverse effect of single status became smaller over time; male patients obtained survival benefits from married status with a longer period of time than female patients (i.e., 1983–2012 vs. 2003–2012; Fig. 4C–D). The strength of the negative effect of separated/divorced status showed a downward trend over time; this trend appeared more earlier in male patients than female patients (i.e., 1973–2012 vs. 1993–2012; Fig. 4E–F). Moreover, the effect of separated/divorced status became nonsignificant in female patients during the period of 2003–2012. The strength of the negative effect of widowed status had an upward trend over time; line charts in Figure 4G–H showed a superb consistency of the tendency between male and female patients. Being widowed had an obviously improved magnitude of the influence on survival in both male and female patients between 2003 and 2012. Detailed information were presented in Table S2.

## Discussion

To the best of our knowledge, this is the first study to assess the impact of marital status at diagnosis on survival outcomes and its change over time in patients with NPC. Based on well‐matched data sets and long‐term follow‐up data, this study provides results with high validity and reliability. Being married was suggested to have a protective effect on survival compared to any unmarried status. The change over time in the effect of marital status was more stable in male than female. According to the recent trend of the change, single and widowed NPC patients, but not separated/divorced patients, are identified as high‐risk population. Interestingly, only non‐Hispanic white and Chinese American patients can obtain survival benefit from married status.

Several potential mechanisms may explain the association between married status and survivorship. Firstly, it has been verified that negative life events can induce psychological stress with adverse influence on health 17, 18. According to the social readjustment rating scale created by Holmes and Rahe, the top three factors with strongest psychological‐stimulating intensity are the death of a spouse, divorce and marital separation 19. Moreover, in itself, a diagnosis of cancer can also result in psychological disorders (e.g., despair, depression, anxiety) compared to receiving other diagnoses 20. Therefore, married patients are more likely to have a positive mood, active attitude to buffer psychological pressure than the unmarried. A robust review reported a variety of downstream effects including release of stress hormones (e.g., catecholamine and cortisol), promotion of tumor growth and migration and stimulation of angiogenesis are induced by physiological stressors 21. These factors could exert an adverse effect on prognosis by directly promoting tumor progression, indirectly triggering inflammatory processes and/or suppressing immunological function 22, 23, 24. Secondly, the spousal supervision and parenthood may help married individuals to maintain positive life style and good physical health 12, 25. Thus, married cancer patients appear more likely to present with earlier stages of tumors at time of diagnosis and accept definitive treatment than their unmarried counterparts 5, 12. Thirdly, with emotional and financial support from spouse/children/close relatives, patients are better able to tolerate radical therapies and have better compliance to medical recommendations 26. Married patients are more likely to afford health insurance because of economic advantages achieved by financial support; the positive influence of health insurance on survival has been reported in head and neck cancers 27. Family can also encourage access to care and help to understand medical information, which may allow patients to receive more timely treatment at superior, specialized hospitals.

In addition to the aforementioned causal effect, that is, being married is conducive to better prognosis, a selection effect of marital status has recently been reported. Men with better socioeconomic resources are usually regarded as desirable partners to have higher possibility to enter and remain in a marriage. However, the socioeconomic status of women are more likely to change over time 12, which is consistent with the results in our study (Fig. 4). One possible reason is that the employment rate of women has changed from 1973 to 2012 28. Moreover, health condition of the individuals who are chosen as ones’ spouses is evidently an determinant of their widowhood. Thus, positive selection of healthy and resourceful individuals (e.g., high education attainment and/or high income) into marriage is favorable to prolong cancer patients’ survival 29.

Several studies have indicated Asian and Chinese patients with NPC have a survival advantage compared to non‐Hispanic white/black patients, which is mainly attributed to genetic factors 30, 31. It has been reported that 98% Chinese NPC patients have non‐keratinizing disease, while up to 25% American NPC patients have keratinizing disease 1. Moreover, NPC patients from endemic regions (e.g., South China) are generally associated with Epstein–Barr virus (EBV) infection and have the most superior OS and local control because of their high sensitivity to radiotherapy. On the contrary, NPC patients from non‐endemic regions (e.g., North America) have the lowest to moderate survival and an underlying relationship with human papillomavirus (HPV) that usually exists in the population with poor socioeconomic status 1, 27, 31. As an advantage group, non‐Hispanic white American are more likely to access medical resources, wealthy and healthy condition, and lower HPV infection rate. Therefore, marriage‐induced survival benefits is more obvious among non‐Hispanic white NPC patients. Besides, genetic factors (e.g., genes in the epidermal growth factor receptor pathway) may also contribute to the racial differences that may be linked with better prognosis in non‐Hispanic white NPC patients, although the definitive associations between those genetic changes and race have not been established 31. It was noteworthy that marriage had no significant survival benefit in non‐Hispanic black or Hispanic patients. Patients with different races/ethnicities have different backgrounds in many respects, such as hereditary characteristics, customs and habits, life styles and dietary differences, which may interact with the impact of marital status on survival to some extent 32. For instance, a previous study reported that non‐Hispanic black Americans younger than 20 years‐old have a higher incidence of NPC compared to other races 33. Moreover, psychosocial effects are induced via a chronic, persistent progress 21, 34. Thus, although we excluded juvenile patients, young black patients may still obtain less benefit from their newly‐married status because of their relatively high‐risk and short duration of marriage. Hispanic ethnicity was recorded independently of race in the SEER database, so patients with Hispanic ethnicity could be of any race. In our study, “other races/ethnicities” subgroup included patients with mixed races, and marriage had a nonsignificant effect on survival in this subgroup (Fig. S1). Therefore, the ambiguous classification of race for patients with Hispanic ethnicity may result in bias and error. Besides, non‐Hispanic black and Hispanic groups only had the sample size of 174 and 177 patients (386 and 254 patients) and represented 6.0% and 6.1% (13.3% and 8.7%) of the total married (unmarried) cases. Thus, results for these groups could be affected by small sample sizes. Nevertheless, this study provides an important indication that a survival advantage related to marriage exists for non‐Hispanic white and Chinese American patients with NPC.

Several limitations must be taken into account. Firstly, a number of essential medical data were not recorded in the SEER database, such as chemotherapy regimen and biological/molecular markers (e.g., EBV‐DNA titer). These factors can sway medical decisions and have significant influence on the survival outcomes in patients with NPC 35, 36. Second, in addition to married status, social support is also closely related to income, insurance status, level of education and residence (e.g., rural or urban). Socioeconomic intervention targeted to vulnerable patients, such as the unmarried, has shown great potential as an adjuvant therapy in other malignancies 5, 11. Although this study does not include comprehensive socioeconomic factors, the conclusions indicate some possible policy implications. For instance, health care workers may take advantage of their ability to attend to single/widowed cancer patients, take greater care to ensure that they can follow‐up the treatment plans and health checks, and produce targeted courses in how to take better care of their own health. Last but not least, this study regarded the marital status at diagnosis as an unalterable status. However, patients may experience changes in marital status after registration or during treatment. As shown in Figure 2A and F, the difference in 5‐year CSS/OS (8.5% and 10.3%) was smaller than the difference in 3‐year CSS/OS (8.8% and 10.6%) between married and unmarried patients. That is, those who are married and die early have a larger probability of still being married at time of death, whereas those with longer follow‐up time have larger chance of having changed their marital status. This rough analysis indirectly suggested that the protective effect of being married is reduced over time possibly due to changes in marital status which are not accounted for. We also ignored the effects of satisfaction and quality of marriage, which have significant influence on health conditions 37.

## Conclusion

Married status had a protective effect on CSS/OS compared to any unmarried status, although only non‐Hispanic white and Chinese American patients can obtain the marriage‐induced survival benefits. Single and widowed NPC patients, but not separated/divorced patients, are regarded as high‐risk population. Future studies are needed to developed appropriate socioeconomic interventions targeted to high‐risk patients.

## Conflict of Interest

The authors declare that they have no competing interests.

## Supporting information


**Table S1.** Baseline characteristics of NPC patients in the three matched cohorts according to marital status.
**Table S2.** Change over time in the effect of marital status on CSS and OS.
**Figure S1.** Kaplan–Meier survival curves in patients with NPC of other races/ethnicities. Survival curves for cause‐specific survival (A) and overall survival (B) were stratified by marital status. Other races/ethnicities include American Indian/Alaska Native, Asian/Pacific Islander, and unknown. HR, hazard ratio; CI, confidence interval; No., number.

## References

[1] Wei, W. I. , and J. S. Sham . 2005 Nasopharyngeal carcinoma. Lancet 365:2041–2054. 15950718 10.1016/S0140-6736(05)66698-6

[2] Karnell, L. H. , A. J. Christensen , E. L. Rosenthal , J. S. Magnuson , and G. F. Funk . 2007 Influence of social support on health‐related quality of life outcomes in head and neck cancer. Head Neck 29:143–146. 17111431 10.1002/hed.20501

[3] Breitbart, W. , and J. Holland . 1988 Psychosocial aspects of head and neck cancer. Semin. Oncol. 15:61–69. 3344444

[4] Manzoli, L. , P. Villari , and M. P. G. A. Boccia . 2007 Marital status and mortality in the elderly: A systematic review and meta‐analysis. Soc Sci Med 64:77–94. 17011690 10.1016/j.socscimed.2006.08.031

[5] Aizer, A. A. , M. H. Chen , E. P. McCarthy , M. L. Mendu , S. Koo , T. J. Wilhite , et al. 2013 Marital status and survival in patients with cancer. J. Clin. Oncol. 31:3869–3876. 24062405 10.1200/JCO.2013.49.6489 PMC4878087

[6] Krongrad, A. , H. Lai , M. A. Burke , K. Goodkin , and S. Lai . 1996 Marriage and mortality in prostate cancer. J Urol. 156:1696–1670. 8863573

[7] Mahdi, H. , S. Kumar , A. R. Munkarah , M. Abdalamir , M. Doherty , and R. Swensen . 2013 Prognostic impact of marital status on survival of women with epithelial ovarian cancer. Psychooncology 22:83–88. 21919121 10.1002/pon.2058

[8] Sammon, J. D. , M. Morgan , O. Djahangirian , Q. D. Trinh , M. Sun , K. R. Ghani , et al. 2012 Marital status: a gender‐independent risk factor for poorer survival after radical cystectomy. BJU Int. 110:1301–1309. 22449122 10.1111/j.1464-410X.2012.10993.x

[9] Jatoi, A. , P. Novotny , S. Cassivi , M. M. Clark , D. Midthun , C. A. Patten , et al. 2007 Does marital status impact survival and quality of life in patients with non‐small cell lung cancer? Observations from the mayo clinic lung cancer cohort. Oncologist. 12:1456–1463. 18165623 10.1634/theoncologist.12-12-1456

[10] Greenberg, E. R. , C. G. Chute , T. Stukel , J. A. Baron , D. H. Freeman , J. Yates , et al. 1988 Social and economic factors in the choice of lung cancer treatment. A population‐based study in two rural states. N. Engl. J. Med. 318:612–617. 2830514 10.1056/NEJM198803103181006

[11] Inverso, G. , B. A. Mahal , A. A. Aizer , R. B. Donoff , N. G. Chau , and R. I. Haddad . 2015 Marital status and head and neck cancer outcomes. Cancer 121:1273–1278. 25524565 10.1002/cncr.29171

[12] Kravdal, H. , and A. Syse . 2011 Changes over time in the effect of marital status on cancer survival. BMC Public Health 11:804. 21999466 10.1186/1471-2458-11-804 PMC3206482

[13] National Cancer Institute . Surveillance Research Program, Surveillance Systems Branch. Surveillance, epidemiology, and end results (SEER) program. Number of persons by race and Hispanic ethnicity for SEER participants (2010 census data). Available at: http://seer.cancer.gov/registries/data.html (accessed June 21, 2017).

[14] Sun Yat‐sen University Cancer Center, Guangzhou . 2017 Research Data Deposit. Available at: http://www.researchdata.org.cn/ (accessed June 21, 2017).

[15] Neyeloff, J. L. , S. C. Fuchs , and L. B. Moreira . 2012 Meta‐analyses and Forest plots using a microsoft excel spreadsheet: step‐by‐step guide focusing on descriptive data analysis. BMC Res. Notes 5:52. 22264277 10.1186/1756-0500-5-52 PMC3296675

[16] Sturmer, T. , M. Joshi , R. J. Glynn , J. Avorn , K. J. Rothman , and S. Schneeweiss . 2006 A review of the application of propensity score methods yielded increasing use, advantages in specific settings, but not substantially different estimates compared with conventional multivariable methods. J. Clin. Epidemiol. 59:437–447. 16632131 10.1016/j.jclinepi.2005.07.004 PMC1448214

[17] Phillips, A. C. , D. Carroll , and G. Der . 2015 Negative life events and symptoms of depression and anxiety: stress causation and/or stress generation. Anxiety Stress Coping 28:357–371. 25572915 10.1080/10615806.2015.1005078 PMC4772121

[18] Moreno‐Smith, M. , S. K. Lutgendorf , and A. K. Sood . 2010 Impact of stress on cancer metastasis. Future Oncol. 6:1863–1881. 21142861 10.2217/fon.10.142 PMC3037818

[19] Holmes, T. H. , and R. H. Rahe . 1967 The social readjustment rating scale. J. Psychosom. Res. 11:213–218. 6059863 10.1016/0022-3999(67)90010-4

[20] Kaiser, N. C. , N. Hartoonian , and J. E. Owen . 2010 Toward a cancer‐specific model of psychological distress: population data from the 2003–2005 National Health Interview Surveys. J. Cancer Surviv. 4:291–302. 20213535 10.1007/s11764-010-0120-3

[21] Antoni, M. H. , S. K. Lutgendorf , S. W. Cole , F. S. Dhabhar , S. E. Sephton , P. G. McDonald , et al. 2006 The influence of bio‐behavioural factors on tumour biology: pathways and mechanisms. Nat. Rev. Cancer 6:240–248. 16498446 10.1038/nrc1820 PMC3146042

[22] Miller, G. E. , S. Cohen , and A. K. Ritchey . 2002 Chronic psychological stress and the regulation of pro‐inflammatory cytokines: a glucocorticoid‐resistance model. Health Psychol. 21:531–541. 12433005 10.1037//0278-6133.21.6.531

[23] Levy, S. M. , R. B. Herberman , T. Whiteside , K. Sanzo , J. Lee , and J. Kirkwood . 1990 Perceived social support and tumor estrogen/progesterone receptor status as predictors of natural killer cell activity in breast cancer patients. Psychosom. Med. 52:73–85. 2305024 10.1097/00006842-199001000-00006

[24] Reiche, E. M. , S. O. Nunes , and H. K. Morimoto . 2004 Stress, depression, the immune system, and cancer. Lancet Oncol. 5:617–625. 15465465 10.1016/S1470-2045(04)01597-9

[25] Kravdal, O. 2003 Children, family and cancer survival in Norway. Int. J. Cancer 105:261–266. 12673689 10.1002/ijc.11071

[26] DiMatteo, M. R. , H. S. Lepper , and T. W. Croghan . 2000 Depression is a risk factor for noncompliance with medical treatment: meta‐analysis of the effects of anxiety and depression on patient adherence. Arch. Intern. Med. 160:2101–2107. 10904452 10.1001/archinte.160.14.2101

[27] Xu, C. , Y. P. Chen , X. Liu , L. L. Tang , L. Chen , Y. P. Mao , et al. 2017 Socioeconomic factors and survival in patients with non‐metastatic head and neck squamous cell carcinoma. Cancer Sci. 108:1253–1262. 28383806 10.1111/cas.13250 PMC5480066

[28] Sweeney, M. M. 2002 Two decades of family change: the shifting economic foundations of marriage. Am. Sociol. Rev. 67:132–147.

[29] Syse, A. , and T. H. Lyngstad . 2017 In sickness and in health: the role of marital partners in cancer survival. SSM Popul Health. 3:99–110. 10.1016/j.ssmph.2016.12.007 PMC5769016 29349208

[30] Sun, L. M. , C. I. Li , E. Y. Huang , and T. L. Vaughan . 2007 Survival differences by race in nasopharyngeal carcinoma. Am. J. Epidemiol. 165:271–278. 17090616 10.1093/aje/kwk008

[31] Wang, Y. , Y. Zhang , and S. Ma . 2013 Racial differences in nasopharyngeal carcinoma in the United States. Cancer Epidemiol. 37:793–802. 24035238 10.1016/j.canep.2013.08.008 PMC3851929

[32] Yong, S. K. , T. C. Ha , M. C. Yeo , V. Gaborieau , J. D. McKay , and J. Wee . 2017 Associations of lifestyle and diet with the risk of nasopharyngeal carcinoma in Singapore: a case‐control study. Chin. J. Cancer. 36:3. 28063457 10.1186/s40880-016-0174-3 PMC5219694

[33] Richey, L. M. , A. F. Olshan , J. George , C. G. Shores , A. M. Zanation , T. Cannon , et al. 2006 Incidence and survival rates for young blacks with nasopharyngeal carcinoma in the United States. Arch. Otolaryngol. Head Neck Surg. 132:1035–1040. 17043247 10.1001/archotol.132.10.1035

[34] McEwen, B. S. 2007 Physiology and neurobiology of stress and adaptation: central role of the brain. Physiol. Rev. 87:873–904. 17615391 10.1152/physrev.00041.2006

[35] Liu, N. , N. Y. Chen , R. X. Cui , W. F. Li , Y. Li , R. R. Wei , et al. 2012 Prognostic value of a microRNA signature in nasopharyngeal carcinoma: a microRNA expression analysis. Lancet Oncol. 13:633–641. 22560814 10.1016/S1470-2045(12)70102-X

[36] Leung, S. F. , A. T. Chan , B. Zee , B. Ma , L. Y. Chan , P. J. Johnson , et al. 2003 Pretherapy quantitative measurement of circulating Epstein‐Barr virus DNA is predictive of posttherapy distant failure in patients with early‐stage nasopharyngeal carcinoma of undifferentiated type. Cancer 98:288–291. 12872347 10.1002/cncr.11496

[37] Jaremka, L. M. , R. Glaser , W. B. Malarkey , and J. K. Kiecolt‐Glaser . 2013 Marital distress prospectively predicts poorer cellular immune function. Psychoneuroendocrinology. 38:2713–2719. 23880114 10.1016/j.psyneuen.2013.06.031 PMC3812415

