# Supplementary material for: Impact of marital status at diagnosis on survival and its change over time between 1973 and 2012 in patients with nasopharyngeal carcinoma: a propensity score‐matched analysis
Source: Cancer Med. 2017 Oct 16;6(12):3040–51. doi: 10.1002/cam4.1232 (PMC5727244; doi:10.1002/cam4.1232)
Supplement: Supplementary file 1 — Table S1. Baseline characteristics of NPC patients in the three matched cohorts according to marital status. Table S2. Change over time in the effect of marital status on CSS and OS. Figure S1. Kaplan–Meier survival curves in patients with NPC of other races/ethnicities. Survival curves for cause‐specific survival (A) and overall survival (B) were stratified by marital status. Other races/ethnicities include American Indian/Alaska Native, Asian/Pacific Islander, and unknown. HR, hazard ratio; CI, confidence interval; No., number. [file CAM4-6-3040-s001.doc]

**Table S1. Baseline characteristics of NPC patients in the three matched cohorts according to marital status**

| Characteristic | Matched cohort 1 | | |  | Matched cohort 2 | | |  | Matched cohort 3 | | |
| --- | --- | --- | --- | --- | --- | --- | --- | --- | --- | --- | --- |
|  | Married no. (%) ^c^ | Single no. (%) ^c^ | *P* |  | Married no. (%) ^c^ | S/D no. (%) ^c^ | *P* |  | Married no. (%) ^c^ | Widowed no. (%) ^c^ | *P* |
| Sample size | 1521 | 1521 | - |  | 754 | 754 |  |  | 606 | 606 | - |
| Age at diagnosis |  |  | 0.238 |  |  |  | 0.748 |  |  |  | 0.764 |
| ≤ 50 years | 884 (58.1) | 916 (60.2) |  |  | 279 (37.0) | 273 (36.2) |  |  | 24 (4.0) | 22 (3.6) |  |
| > 50 years | 637 (41.9) | 605 (39.8) |  |  | 475 (63.0) | 481 (63.8) |  |  | 582 (96.0) | 584 (96.4) |  |
| Gender |  |  | 0.521 |  |  |  | 0.303 |  |  |  | 0.902 |
| Male | 1079 (70.9) | 1095 (72.0) |  |  | 505 (67.0) | 486 (64.5) |  |  | 196 (32.3) | 198 (32.7) |  |
| Female | 442 (29.1) | 426 (28.0) |  |  | 249 (33.0) | 268 (35.5) |  |  | 410 (67.7) | 408 (67.3) |  |
| Race/ethnicity |  |  | < 0.001 |  |  |  | < 0.001 |  |  |  | 0.002 |
| Non-Hispanic white | 699 (46.0) | 544 (35.8) |  |  | 448 (59.4) | 426 (56.5) |  |  | 355 (58.6) | 330 (54.5) |  |
| Non-Hispanic black | 110 (7.2) | 248 (16.3) |  |  | 48 (6.4) | 75 (9.9) |  |  | 30 (5.0) | 63 (10.4) |  |
| Hispanic | 92 (6.0) | 153 (10.1) |  |  | 35 (4.6) | 56 (7.4) |  |  | 33 (5.4) | 42 (6.9) |  |
| Chinese | 325 (21.4) | 252 (16.6) |  |  | 109 (14.5) | 66 (8.8) |  |  | 93 (15.3) | 70 (11.6) |  |
| Other ^a^ | 295 (19.4) | 324 (21.3) |  |  | 114 (15.1) | 131 (17.4) |  |  | 95 (15.7) | 101 (16.7) |  |
| Registry region ^b^ |  |  | 0.269 |  |  |  | 0.586 |  |  |  | 0.994 |
| West | 1004 (66.0) | 1040 (68.4) |  |  | 466 (61.8) | 475 (63.0) |  |  | 383 (63.2) | 383 (63.2) |  |
| Northeast | 182 (12.0) | 190 (12.5) |  |  | 108 (14.3) | 110 (14.6) |  |  | 76 (12.5) | 74 (12.2) |  |
| North central | 206 (13.5) | 177 (11.6) |  |  | 122 (16.2) | 104 (13.8) |  |  | 102 (16.8) | 105 (17.3) |  |
| South | 129 (8.5) | 114 (7.5) |  |  | 58 (7.7) | 65 (8.6) |  |  | 45 (7.4) | 44 (7.3) |  |
| SEER historic stage |  |  | 0.286 |  |  |  | 0.646 |  |  |  | 0.826 |
| Localized | 144 (9.5) | 144 (9.5) |  |  | 69 (9.2) | 61 (8.1) |  |  | 75 (12.4) | 77 (12.7) |  |
| Regional | 805 (52.9) | 773 (50.8) |  |  | 403 (53.4) | 426 (56.5) |  |  | 329 (54.3) | 322 (53.1) |  |
| Distant | 462 (30.4) | 508 (33.4) |  |  | 227 (30.1) | 218 (28.9) |  |  | 152 (25.1) | 148 (24.4) |  |
| Unstaged | 110 (7.2) | 96 (6.3) |  |  | 55 (7.3) | 49 (6.5) |  |  | 50 (8.3) | 59 (9.7) |  |
| Histological type |  |  | 0.605 |  |  |  | 0.069 |  |  |  | 0.793 |
| Keratinizing | 562 (36.9) | 546 (35.9) |  |  | 331 (43.9) | 351 (46.6) |  |  | 280 (46.2) | 289 (47.7) |  |
| Differentiated non‑keratinizing | 248 (16.3) | 229 (15.1) |  |  | 102 (13.5) | 98 (13.0) |  |  | 75 (12.4) | 64 (10.6) |  |
| Undifferentiated non‑keratinizing | 319 (21.0) | 332 (21.8) |  |  | 138 (18.3) | 102 (13.5) |  |  | 103 (17.0) | 105 (17.3) |  |
| Other | 392 (25.8) | 414 (27.2) |  |  | 183 (24.3) | 203 (26.9) |  |  | 148 (24.4) | 148 (24.4) |  |
| Year of diagnosis |  |  | 0.823 |  |  |  | 0.581 |  |  |  | 0.074 |
| 1973 to 1982 | 141 (9.3) | 136 (8.9) |  |  | 77 (10.2) | 74 (9.8) |  |  | 107 (17.7) | 86 (14.2) |  |
| 1983 to 1992 | 194 (12.8) | 186 (12.2) |  |  | 102 (13.5) | 107 (14.2) |  |  | 116 (19.1) | 125 (20.6) |  |
| 1993 to 2002 | 443 (29.1) | 466 (30.6) |  |  | 198 (26.3) | 219 (29.0) |  |  | 167 (27.6) | 201 (33.2) |  |
| 2003 to 2012 | 743 (48.8) | 733 (48.2) |  |  | 377 (50.0) | 354 (46.9) |  |  | 216 (35.6) | 194 (32.0) |  |
| Treatment |  |  | 0.716 |  |  |  | 0.941 |  |  |  | 0.574 |
| Surgery and/or RT | 1302 (85.6) | 1309 (86.1) |  |  | 649 (86.1) | 650 (86.2) |  |  | 518 (85.5) | 511 (84.3) |  |
| No definitive treatment | 219 (14.4) | 212 (13.9) |  |  | 105 (13.9) | 104 (13.8) |  |  | 88 (14.5) | 95 (15.7) |  |

NPC, nasopharyngeal carcinoma; no., number; S/D, separated/divorced; RT, radiotherapy; SEER, Surveillance, Epidemiology, and End Results Program

^a^ ‘Other’ includes American Indian/Alaska Native, Asian/Pacific Islander, and unknown

^b^ ‘West’ includes Seattle-Puget Sound, Greater California, San Francisco-Oakland, San Jose-Monterey, Los Angeles, Utah, New Mexico, Alaska, and Hawaii. ‘Northeast’ includes Connecticut and New Jersey. ‘North central’ includes Iowa and Detroit. ‘South’ includes Kentucky, Atlanta, Rural Georgia, Greater Georgia, and Louisiana

^c^ Percentages may not add up to 100 because of rounding

**Table** S2. Change over time in the effect of marital status on CSS and OS

| 1-to-1 matched cohorts | CSS | | |  | OS | |
| --- | --- | --- | --- | --- | --- | --- |
|  | HR | 95% CI | |  | HR | 95% CI |
| Unmarried vs. married (Ref.) | | | | | | |
| Male |  |  | |  |  |  |
| 1973-1982 | 1.20 | 0.92-1.56 | |  | 1.19 | 0.93-1.53 |
| 1983-1992 | 1.31 | 1.02-1.67 | |  | 1.39 | 1.11-1.74 |
| 1993-2002 | 1.35 | 1.11-1.64 | |  | 1.39 | 1.16-1.66 |
| 2003-2012 | 1.24 | 1.05-1.46 | |  | 1.31 | 1.13-1.53 |
| Female |  |  | |  |  |  |
| 1973-1982 | 1.23 | 0.86-1.73 | |  | 1.23 | 0.90-1.69 |
| 1983-1992 | 1.63 | 1.17-2.28 | |  | 1.69 | 1.24-2.31 |
| 1993-2002 | 1.31 | 1.02-1.68 | |  | 1.40 | 1.12-1.76 |
| 2003-2012 | 1.86 | 1.45-2.38 | |  | 1.76 | 1.41-2.19 |
| Single vs. married (Ref.) | | | | | | |
| Male |  | |  |  |  |  |
| 1973-1982 | 0.98 | | 0.69-1.41 |  | 1.03 | 0.75-1.43 |
| 1983-1992 | 1.39 | | 0.99-1.93 |  | 1.33 | 1.00-1.78 |
| 1993-2002 | 1.27 | | 1.00-1.61 |  | 1.24 | 1.01-1.53 |
| 2003-2012 | 1.38 | | 1.12-1.71 |  | 1.38 | 1.13-1.67 |
| Female |  | |  |  |  |  |
| 1973-1982 | 1.32 | | 0.69-2.53 |  | 1.30 | 0.72-2.34 |
| 1983-1992 | 1.05 | | 0.58-1.90 |  | 0.88 | 0.51-1.52 |
| 1993-2002 | 1.07 | | 0.74-1.54 |  | 1.13 | 0.81-1.58 |
| 2003-2012 | 1.54 | | 1.05-2.26 |  | 1.55 | 1.09-2.20 |
| Separate/divorced vs. married (Ref.) | | | | | | |
| Male |  | |  |  |  |  |
| 1973-1982 | 2.03 | | 1.23-3.37 |  | 1.97 | 1.27-3.07 |
| 1983-1992 | 1.42 | | 0.92-2.21 |  | 1.34 | 0.91-1.97 |
| 1993-2002 | 1.21 | | 0.86-1.70 |  | 1.26 | 0.93-1.71 |
| 2003-2012 | 1.39 | | 1.04-1.86 |  | 1.40 | 1.08-1.81 |
| Female |  | |  |  |  |  |
| 1973-1982 | 0.85 | | 0.42-1.72 |  | 0.96 | 0.52-1.77 |
| 1983-1992 | 1.29 | | 0.67-2.50 |  | 1.19 | 0.67-2.11 |
| 1993-2002 | 1.79 | | 1.10-2.92 |  | 1.89 | 1.21-2.95 |
| 2003-2012 | 1.22 | | 0.75-1.98 |  | 1.11 | 0.72-1.69 |
| Widowed vs. married (Ref.) | | | | | | |
| Male |  | |  |  |  |  |
| 1973-1982 | 0.85 | | 0.44-1.63 |  | 0.80 | 0.46-1.38 |
| 1983-1992 | 1.32 | | 0.76-2.30 |  | 1.46 | 0.90-2.36 |
| 1993-2002 | 1.23 | | 0.75-2.02 |  | 1.31 | 0.85-2.02 |
| 2003-2012 | 1.96 | | 1.18-3.24 |  | 1.94 | 1.23-3.07 |
| Female |  | |  |  |  |  |
| 1973-1982 | 0.97 | | 0.62-1.51 |  | 1.01 | 0.68-1.49 |
| 1983-1992 | 1.38 | | 0.90-2.12 |  | 1.50 | 1.04-2.17 |
| 1993-2002 | 1.09 | | 0.77-1.55 |  | 1.26 | 0.93-1.69 |
| 2003-2012 | 2.15 | | 1.47-3.15 |  | 2.20 | 1.58-3.06 |

CSS, cause-specific survival; OS, overall survival; Ref., reference; HR, hazard ratio; CI, confidence interval


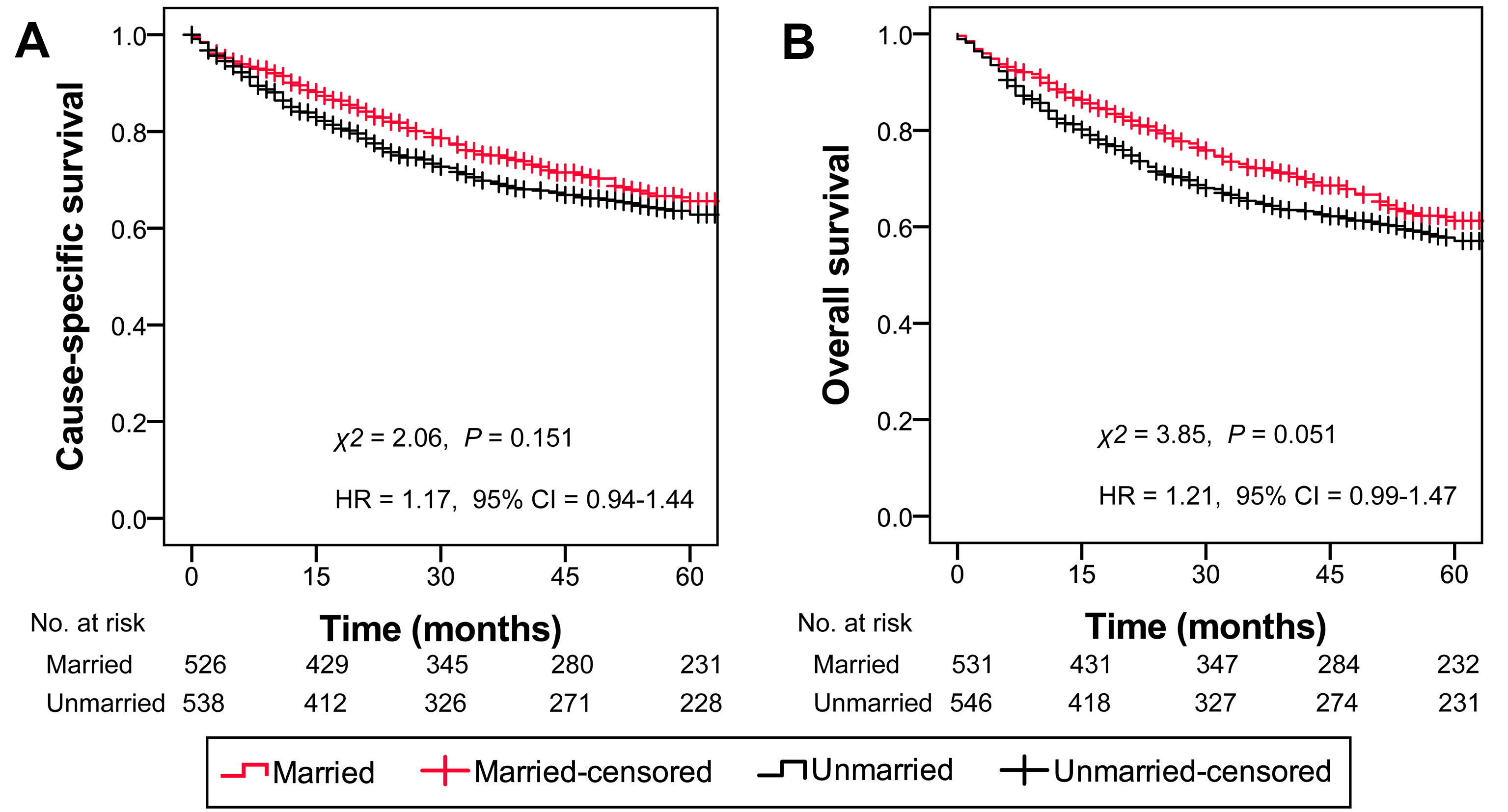


**Fig. S1.** Kaplan-Meier survival curves in patients with NPC of other races/ethnicities. Survival curves for cause-specific survival (**A**) and overall survival (**B**) were stratified by marital status. Other races/ethnicities include American Indian/Alaska Native, Asian/Pacific Islander, and unknown. HR, hazard ratio; CI, confidence interval; No., number.
